# Effects of chronic caffeine intake and withdrawal on neural activity assessed via resting-state functional magnetic resonance imaging in mice

**DOI:** 10.1016/j.heliyon.2022.e11714

**Published:** 2022-11-19

**Authors:** Mitsuki Rikitake, Sachiko Notake, Karen Kurokawa, Junichi Hata, Fumiko Seki, Yuji Komaki, Hinako Oshiro, Naoki Kawaguchi, Yawara Haga, Daisuke Yoshimaru, Ken Ito, Hirotaka James Okano

**Affiliations:** a Graduate School of Human Health Sciences, Tokyo Metropolitan University, Tokyo, Japan; b The Jikei University Graduate School of Medicine, Division of Regenerative Medicine, Tokyo, Japan; c Central Institute for Experimental Animals, Live Imaging Center, Kanagawa, Japan

**Keywords:** Caffeine, Resting-state fMRI, Neural network, Independent component analysis, Functional connectivity analysis

## Abstract

Caffeine is a psychoactive substance that not only improves wakefulness, but also slows the cognitive decline caused by aging. However, at present, there are no reports about the effects of caffeine withdrawal, including headaches and changes in brain functional networks (nerve activity). Headache may occur approximately 24 h after discontinuing caffeine intake in chronic caffeine drinkers.

The current study aimed to examine the brain functional activity via resting-state functional magnetic resonance imaging in chronically caffeinated and decaffeinated groups to investigate changes in brain activity caused by caffeine.

C57BL/6J mice were included in the analysis, and they underwent 9.4-T ultrahigh-field magnetic resonance imaging. The mice were classified into the control, chronic caffeinated, and caffeine withdrawal grsoups. Mice were divided into three groups: 1) not exposed to caffeine (control); 2) treated with caffeine at a concentration of 0.3 mg/mL for 4 weeks (chronic caffeinated); and 3) treated as before with caffeine and withdrawn from caffeine for 24 h. After the three groups were examined, functional connectivity matrices were calculated using brain imaging analysis tools, and independent component analysis was performed.

The results showed that caffeine administration activated neural activity areas in the stress response system. Furthermore, 24h after caffeine withdrawal, the results showed an increase in pain-related neural activity. In addition, caffeine administration was shown to activate the dentate gyrus, one of the hippocampal regions, and to decrease the neural activity in the olfactory bulb and anterior cingulate cortex.

In the current research, the neural activity of specific brain regions changed after chronic caffeine administration and withdrawal.

## 1 Introduction

Caffeine is an organic compound commonly used in our daily lives and is mainly found in coffee, tea, and chocolate. It is frequently utilized as a psychoactive substance that stimulates the central nervous system [1]. Caffeine is an antagonist of adenosine receptors, which biphasically modulates the release of different neurotransmitters, namely, glutamate and dopamine, to either bolster or normalize neuronal function depending on the ongoing activity [2]. This compound increases alertness, reduces symptoms of sleep deprivation, delays the onset of neurological conditions such as Alzheimer’s disease and slows the decline of cognitive abilities caused by aging [3].

Caffeine intake diminishes the reaction times for tasks correlated with attention and working memory and improves the accuracy of numerical calculations [4]. Caffeine can also improve motor function. In a previous study, the average time for three 1500-m runs was approximately 3 s faster after drinking caffeinated coffee than after drinking decaffeinated coffee [5]. Hence, it was a prohibited doping agent among athletes until 2004. In addition, in a study on mice, an analysis of molecular pathways involved in neural processing using orthogonal omics revealed that chronic caffeine administration alters metabolic processes related to lipids and mitochondria and enhances the neuronal activity in response to learning [6]. Chronic caffeine intake has been reported to affect the learning/training-induced transcriptome by significantly increasing the number of regulated genes. Caffeine has different benefits. However, the effect of caffeine on neural activity and the brain regions that are activated remain unclear.

In addition, the discontinuation of caffeine consumption may cause headaches after approximately 24 h among chronic caffeine takers [7]. The American Psychiatric Association has listed caffeine withdrawal symptoms in the Diagnostic and Statistical Manual of Mental Disorders, Fifth Edition.

People who consume 200 mg/day of caffeine for more than 2 weeks continuously experience headache, decreased alertness, and drowsiness within 24 h after the interruption of caffeine intake. These withdrawal symptoms are relieved by reintroducing a smaller amount of caffeine (100 mg) [8]. However, at present, there are no reports about changes in the brain functional networks during caffeine withdrawal, and some aspects of current studies are ambiguous.

Recently, functional magnetic resonance imaging (fMRI), a noninvasive method, has been performed to assess neural activity. In particular, resting-state fMRI (rs-fMRI) has attracted significant attention because of its ability to evaluate the neural activity in the unconscious state.

The neural activity of some brain regions decreases during some types of movements and increases during rest [7, 9]. In addition to the increase in blood oxygenation level-dependent (BOLD) signal, resting-state signals are consistent low-frequency fluctuations in the range of 0.01–0.08 Hz [7, 9]. Approximately 60%–80% of energy is consumed by the whole brain at rest [10].

The default mode network (DMN) is one of the most well-known resting networks. Moreover, it is a neural circuit that is activated if the brain is in a daze, without conscious activity, and that is activated in conjunction with the medial frontal lobe and the posterior cingulate gyrus.

DMN is the most commonly assessed network via rs-fMRI. A previous study conducted in 2004 has shown that the activity in the DMN is significantly reduced in Alzheimer’s disease [11]. Thereafter, there has been a rapid increase in the number of studies on the use of rs-fMRI in central nervous system diseases. Moreover, previous studies have used rs-fMRI to compare regular caffeine drinkers and non-caffeine drinkers [12].

We selected rodents in the current study because the resting-state network evaluated via rs-fMRI is applicable to humans as well as mice and rats because some functional networks of rodents are highly similar to those of humans [13]. In addition, rs-fMRI in rodents cause quite fewer genetic differences between individuals compared than humans when inbred mice are used. Since the rearing environment is almost always similar between different individuals, it is easy to eliminate the effects of individual variations in neural activity attributed to environmental differences [13].

The current study aimed to evaluate changes in brain activity induced by caffeine in a more specific manner in mice. In addition, the benefits of chronic caffeine intake and the changes in brain activity during caffeine withdrawal from the viewpoint of neural activity were analyzed via rs-fMRI.

## 2 Materials and methods

### 2.1 Animal preparation

Male C57BL/6J mice (Charles River Laboratories Japan, Inc., Yokohama-shi, Japan; n = 9 in the control group, n = 14 in the chronic caffeinated group, and n = 17 in the caffeine withdrawal group) were included in the study. The analysis was started when the mice were 7 weeks old. Then, the mice started ingesting caffeine at 16–20 weeks of age, and they were housed (three per cage) under a 12-h light/dark cycle (lights on from 7 am to 7 pm) with free access to food and water.

We selected rodents because they have functional networks, such as the thalamocortical network and DMN, with a highly anatomical similarity to humans on rs-fMRI. Moreover, their brain activity is not easily affected by genetic differences or environmental factors [13]. All procedures were performed per the Laboratory Animal Welfare Act and the Guide for the Care and Use of Laboratory Animals (National Institutes of Health, Bethesda, MD, USA). This study was conducted with the approval of the animal ethics committee (approval number: 2020-024C2) of the Jikei University.

### 2.2 Treatment of animals before rs-fMRI

#### 2.2.1 Surgical and MRI acclimatization training for rs-fMRI

During MRI, the head of each mouse was immobilized to limit movement during wakefulness. In this study, the mice were then fixed at a height where the inferior border of the orbit and the upper border of the external auditory canal could be connected in a straight line, and a head post was surgically attached to the head of each mouse. The 3 × 3-mm and 3-cm-long head post was fixed directly on the cranium of the mouse using dental cement (Sun Medical Company, Ltd., 204610555) that was not affected by magnetization to some degree [14, 15]. To fix the head post on the head with cement after the hair and skin surface were removed, the skin surface was rinsed with saline solution; then, etched gel (Sun Medical Company, Ltd., 204610555) was applied to fill small indentations in the skull to increase the strength of the cement bond [16].

During surgery, the isoflurane (Isoflurane Inhalation Solution 250 mL, 205KOA, Pfizer Inc., New York, NY, the USA) concentration was adjusted to 1.2%–1.8%, and the respiratory rate was modified to 30–40 breaths per minute.

Rs-fMRI was performed under waking conditions. To prevent the effect of long immobilization and noise inherent to MRI on brain activity, mice are often trained before imaging to get them used to rs-fMRI [14, 15]. That is, the mice were exposed to noise generated during fMRI by immobilizing their heads on a special fixation table in the waking state [17] under conditions similar to those of MRI. The training time was 40 min, and each mouse underwent the training for 4 days. To facilitate similar conditions under MRI, the inside of the soundproof box was darkened, and the head was fixed using a fixation platform into which the head post could be inserted. In addition, a scanner sound (approximately 100 dB), which is comparable to the noise generated during fMRI, was played back from a speaker recording. A previous study in which acclimation was conducted under similar conditions reported the stabilization of heart rate and decrease in electromyography signals that may cause head movement [17].

Subsequently, 10-week-old C57BL/6J mice, which are experimental animals maintained by the Jikei University, Tokyo, underwent ultrahigh-field MRI.

#### 2.2.2 Caffeine treatment in mice

After the control group underwent MRI, caffeine (Sigma’s 1,3,7-trimethylxanthine) at a concentration of 0.3 mg/mL was dissolved in the drinking water of mice. The mice drank an average of 5 mL per day [18]. Hence, each mouse received 1.5 mg of caffeine per day. A daily dose of 1.5 mg in mice is equivalent to a daily caffeine intake of approximately 500 mg in humans (approximately five cups of coffee) [18]. After the chronic administration of caffeine for 1 month, MRI was performed again, and the mice treated with caffeine were included in the chronic caffeine group. After imaging, caffeinated water was changed to water. After 24 h, MRI was performed, and the mice were classified into the caffeine withdrawal group. The half-life of caffeine in the blood of adults is approximately 4 h. However, individual differences are observed, which range from 2 to 8 h [16]. The metabolic rate in mice (average body weight = 0.025 kg) is 7.2 times higher than that in humans (average body weight = 68 kg) [19]. Since the average caffeine concentration in humans is reduced to 1/16th in 24 h, mice could lose a lot of caffeine with a withdrawal period of 24 h.

**Figure 1:**
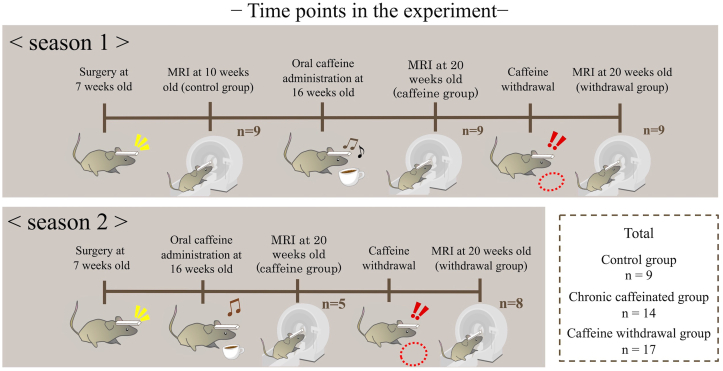
Time points of the experiment in this study. Mice were surgically fitted with head posts at 7 weeks of age. After the control group underwent imaging, the mice consumed caffeine for 4 weeks, and MRI images of the chronic caffeinated group were obtained. Then, after a 24-h withdrawal period, the caffeine withdrawal group underwent MRI. In the second season, caffeine was administered starting at 16 weeks of age, which is similar to the first season, and the control group did not undergo imaging. The rearing environment was similar to that in season 1. During the second season, the number of mice in the chronic caffeine group was reduced due to a problem with MRI.

Figure 1 shows the rough time points of the experiment. After the control group underwent imaging, a series of MRI experiments of caffeine administration and chronic caffeine and caffeine withdrawal was performed on the same individual.

### 2.3 MRI equipment and pulse sequence

To maintain a high signal-to-noise (S/N) ratio [20], we used a 9.4-T Biospec 94/20 MRI (Bruker BioSpin, Ettlingen, Germany), which is a high-field MRI system for animals, and a cryogenic quadrature radio frequency surface probe (CryoProbe; Bruker BioSpin AG, Fällanden, Switzerland). The use of a cryoprobe rather than a volume coil improves the S/N of mouse brain imaging by approximately three-fold [21, 22].

During imaging, the heads of the mice were fixed using a head post. The body was gently wrapped with a paper towel to prevent the mouse from protruding from the bed. MRI was performed for approximately 50 min under waking conditions.

We obtained coronal views by setting the imaging plane perpendicular to the midline and the parietal surface. T2-weighted images were obtained using a rapid acquisition with refocused echo (RARE) sequence (repetition time = 7000 ms, effective echo time = 42 ms, number of averages = 6, RARE factor = 8, scan time = 10 min 30 ms, field-of-view = 15 mm × 12 mm, resolution = 100 μm × 100 μm, matrix = 150 × 120, and slice thickness = 300 μm).

The gradient echo–echo planar imaging acquisition parameters were as follows: repetition time = 1500 ms, effective echo time = 12 ms, number of averages = 1, repetitions = 400, scan time = 10 min, field-of-view = 15 × 12 mm, resolution = 200 × 200 μm, matrix = 75 × 60, and slice thickness = 600 μm. By performing 10-minute fMRI imaging sessions three times in each mouse, the rs-fMRI data increased. The gradient echo method is highly sensitive to magnetic field inhomogeneity and can be combined with echo planar imaging for fast imaging. High-speed imaging can improve the temporal resolution and suppress artifacts such as body motion and breathing [23, 24].

### 2.4 Image analysis methods

#### 2.4.1 Image preprocessing

Since the images obtained via gradient echo–echo planar imaging are significantly affected by magnetic susceptibility, top-up processing was performed using the FMRIB Software Library (FSL, Oxford, the UK) to correct the geometric image distortion [25]. The fMRI images were then preprocessed using a statistical parametric map 12 [26]. All images were processed to be magnified 10-fold in the x, y, and z dimensions to account for the relative size difference between human and rodent brains [27].

The slice timing was corrected because a shift in the BOLD signal acquisition timing may have affected the analysis results. Then, a template using tissue probability maps of the gray matter, white matter, and brain fluid was used to match the spatial coordinates of each mouse [28]. Smoothing using a filter with a full width at a half maximum of 2–3 can stand out the correlation between neural activity and brain regions and can increase the detectability of functional connectivity analysis [29]. Then, CONN (http://www.nitrc.org/projects/conn; The Gabrieli Lab. McGovern Institute for Brain Research, MIT, the USA), software used to analyze functional connectivity, was used.

The BOLD signal handled in rs-fMRI is within extremely low frequencies (0.009–0.1 Hz) [30, 31]. In this study, we extracted the BOLD signal at rest by filtering the 0.009–0.09 Hz band. At the same time, to remove the noise caused by physiological pulsation due to body movement and cerebrospinal fluid, in addition to the neuronal activity linked to hemodynamics, noise reduction processing was performed using the CONN’s a CompCor method.

#### 2.4.2 Neural network analysis

After image preprocessing, independent component analysis (ICA) was conducted to separate the main networks with a strong neural activity from other signal sources [32].

In this study, MELODIC (version 3.15, Christian F. Beckmann, University of Oxford), a part of FSL, was used to perform ICA analysis. The number of components of the brain neural networks to be separated was set to 30. Then, the neural networks in each group that were similar to the DMN and other resting networks were identified.

In addition, functional connectivity analysis was performed to analyze the correlation coefficient of the BOLD signal variation between arbitrarily set regions of interest (ROIs). The total number of ROIs was calculated as (336 × 336 − 336)/2 = 56,280. In this study, we used an atlas composed of 336 ROIs, as in the study of A.E. Dorr et al. [28].

#### 2.4.3 Statistical analysis

In the functional connectivity analysis, the *t*-test between correlation coefficients using the Fisher z-transform by Meng et al. was used to determine significant differences in the correlation coefficients of each group [30, 33]. A p-value of <0.05 was considered significant.

## 3 Results

### 3.1 Networks detected via ICA

**Figure 2:**
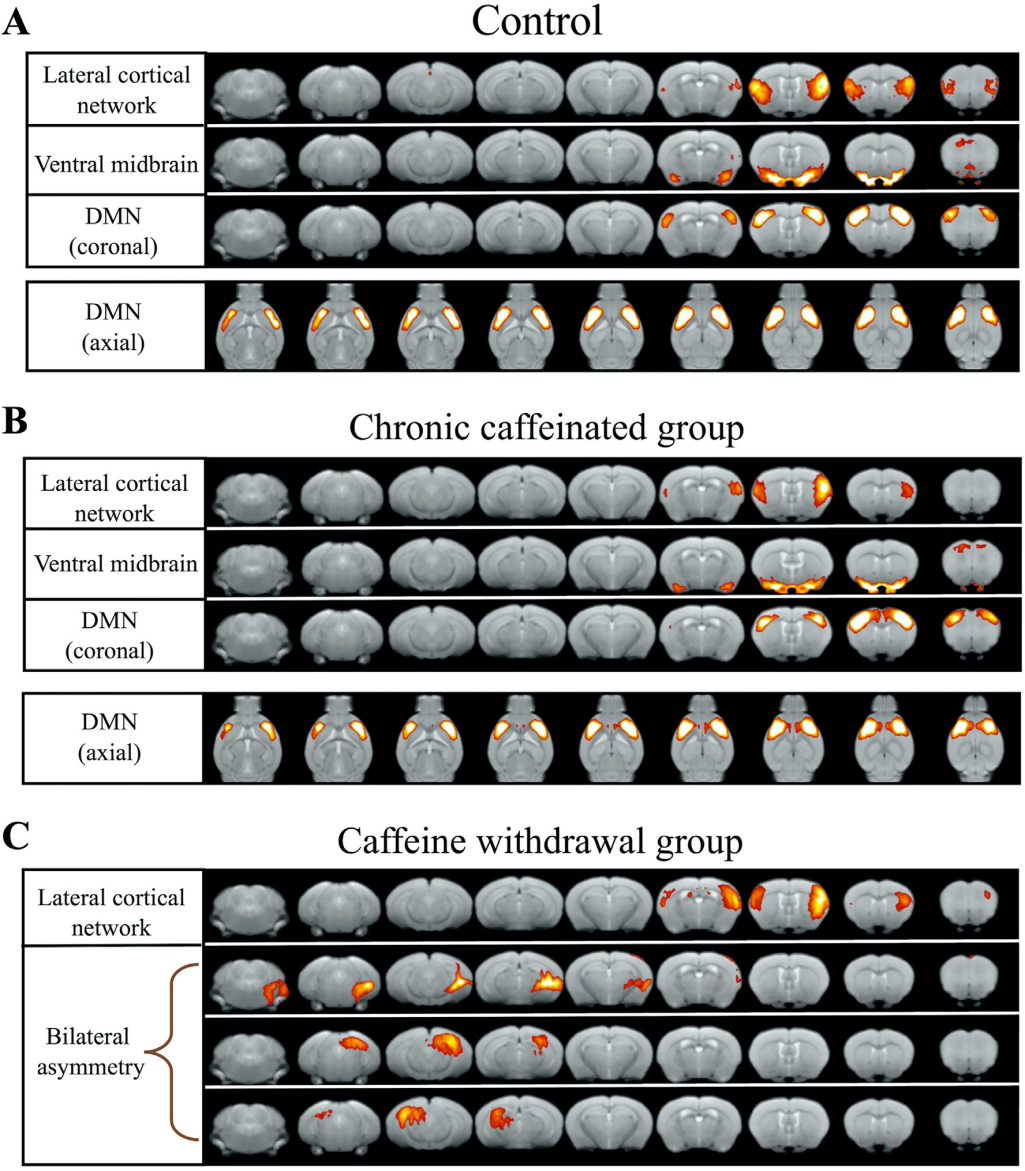
From top to bottom, the ICA analysis results in the control (A), chronic caffeinated group (B), and caffeine withdrawal group (C) are shown in the coronal plane. We arranged the detected networks around the resting-state networks reported in the previous study. The background image is the T2WI of the template image used in this study. For the DMN extracted from the control and chronic caffeine groups, the axial images are lined up at the bottom.

The current study assessed differences in cerebral nerve activity between the control group and the chronic caffeinated and caffeine withdrawal groups. First, ICA was performed on these groups. The number of components of the brain network to be separated was set as 30, and Figure 2 shows the activated regions.

Results showed a network similar to the DMN in the control and chronic caffeinated groups. No differences were observed between the control and chronic caffeine groups regarding the strength of these two networks. However, a DMN-like network was not found in the caffeine withdrawal group. Moreover, the caffeine withdrawal group presented with asymmetrical brain activity. For resting-state networks other than the DMN, we detected networks similar to the lateral cortical network and ventral midbrain.

### 3.2 Neural activity of the whole brain in the matrix analysis

**Figure 3:**
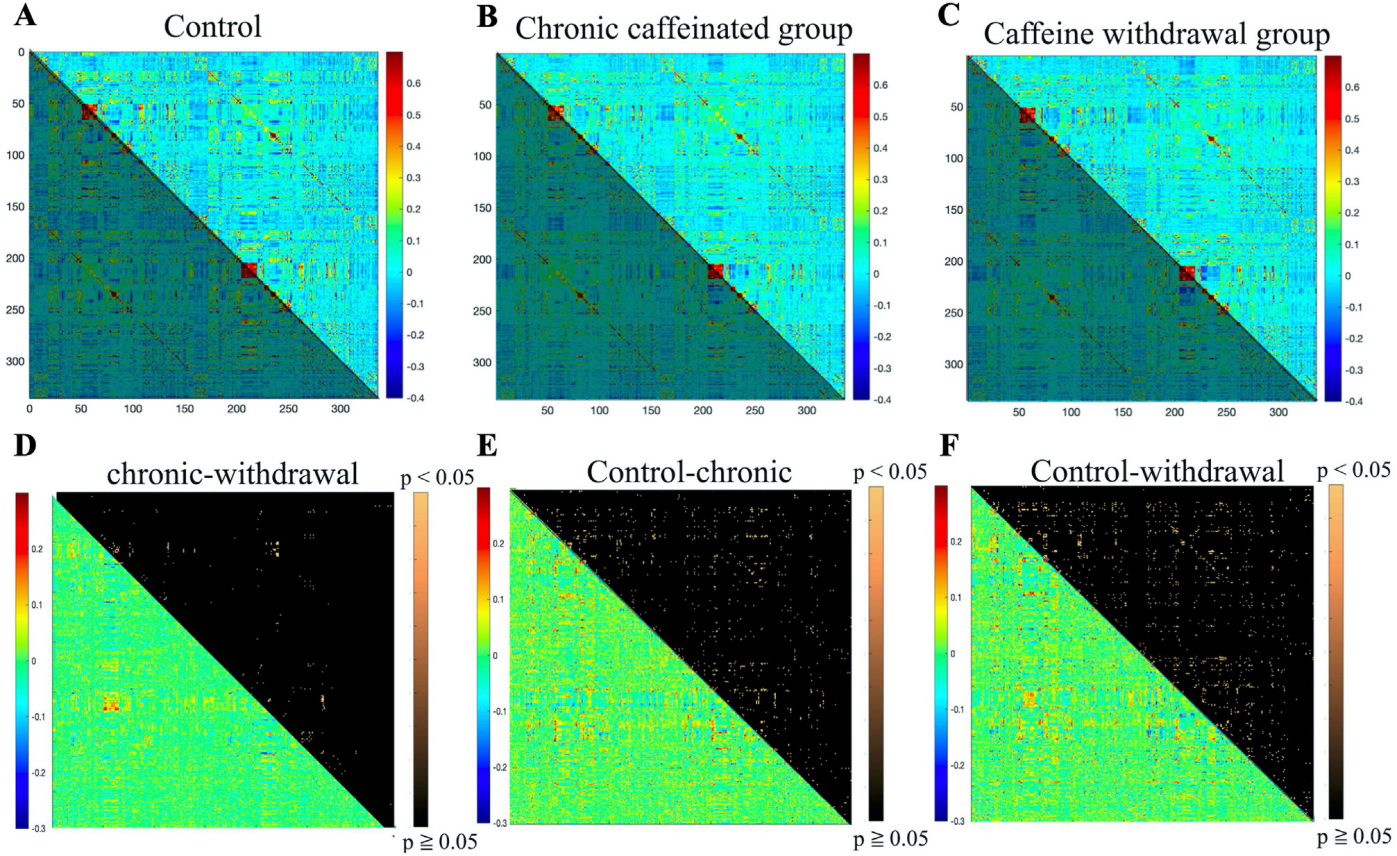
In the upper row, the matrix results of the functional connectivity analysis are shown (from left to right for the control (A), chronic caffeine (B), and caffeine withdrawal groups (C)). The lower triangle of each data has different colors because the data are identical. The vertical and horizontal axes represent 336 brain regions. The color scale indicates the correlation coefficient; red, positive correlation; and blue, negative correlation. In the lower part (From D to F), the statistical methods of Meng and Colleagues were used to determine if there was a significant difference in the correlation coefficients between the “chronic caffeine group vs. caffeine withdrawal group,” “control group vs. chronic caffeine group,” and “control group vs. control group vs. chronic caffeine group” and “control group vs. caffeine withdrawal group” to assess if there was a significant difference in the correlation coefficients (upper triangle). The table area with significant differences is represented by beige color (p < 0.05). By subtracting the absolute values of the correlation coefficients, we examined the differences in brain function with and without caffeine and before and after withdrawal in a more multidimensional manner (lower triangle).

We investigated the neural activity of 336 brain regions. As presented in the upper part of Figure 3, fewer blue areas, which indicated negative correlations, were observed in the chronic caffeine group than in the control group. Compared with the findings in the caffeine withdrawal group, there were few major differences. The three groups exhibited positive correlations in similar areas, indicating that the broad patterns are common.

In addition, with a focus on the areas with significant differences (p < 0.05), a comparison of the chronic caffeine group with the caffeine withdrawal group showed that there were only 197 brain regions that were significantly different. The total number of ROIs was 56,280, indicating that 0.35% of the entire region significantly differed between the groups.

In addition, 901 regions (1.60% of the whole region) significantly differed when the control group was compared to the chronic caffeinated group, and there was a remarkable difference in 1227 regions (2.18% of the whole region) when the control group was compared with the caffeine withdrawal group. Next, we performed a detailed analysis of specific brain regions.

### 3.3 Neural activity of the anterior cingulate cortex in the matrix analysis (neural networks involved in migraine)

In total, 14 regions, primarily the anterior cingulate cortex (ACC) and the somatosensory cortex (SCC), were assessed, and the neural activity of each group was examined.

**Figure 4:**
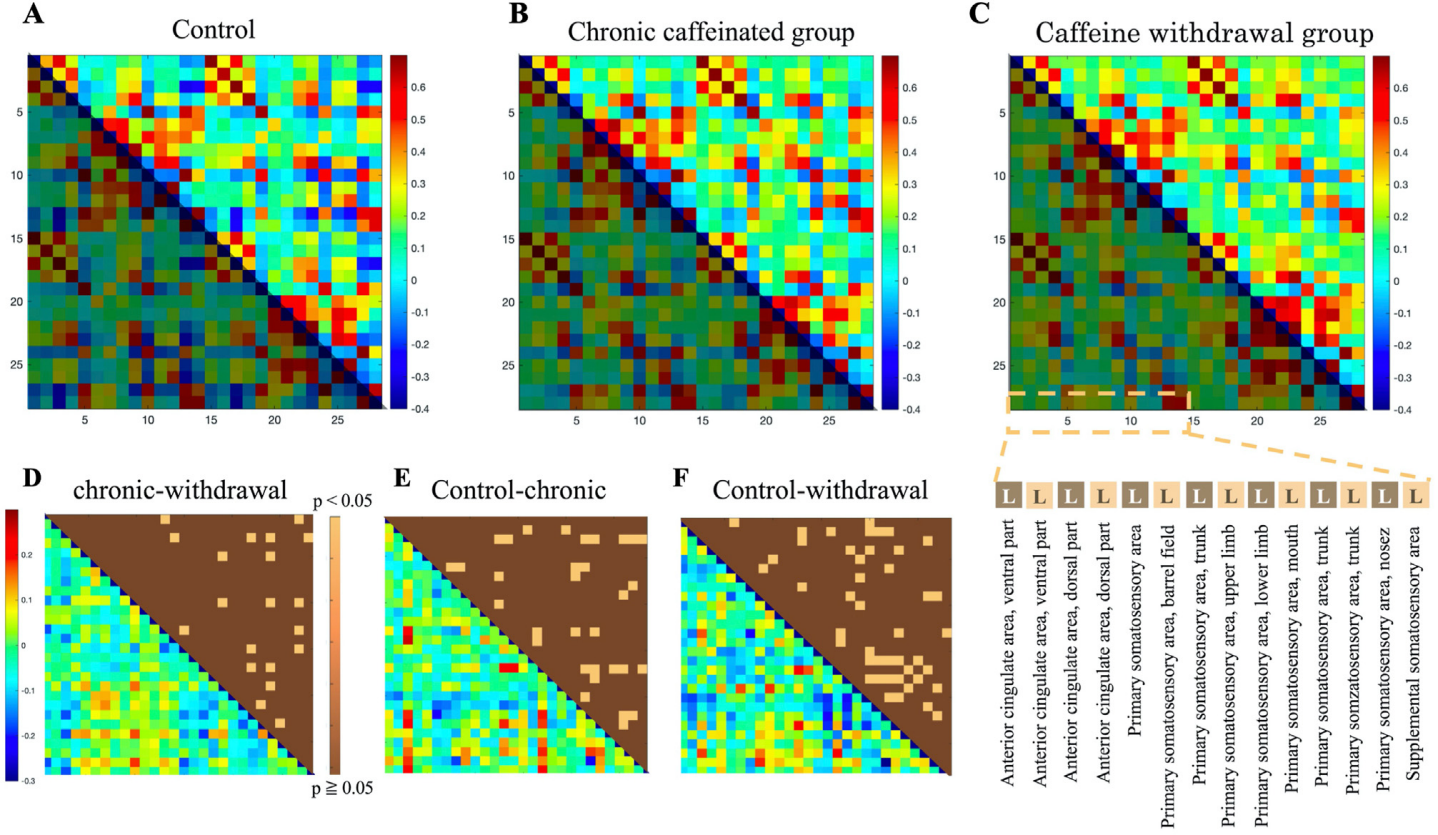
In the upper row, the matrix results of the functional connectivity analysis are shown (from left to right for the control (A), chronic caffeine (B), and caffeine withdrawal groups (C)). The vertical and horizontal axes represent the ACC–SCCs extracted from 336 brain regions. The lower triangle of each data has different colors because they are identical. In the lower part (From D to F), as in Figure 3, the correlation coefficient significance analysis (upper triangle) results and the subtraction of the absolute value of the correlation coefficient (lower triangle) are presented in the bottom row. The details of the extracted region names are shown in the lower right corner. R is omitted because it has the same region name and the same order as L.

**Figure 5:**
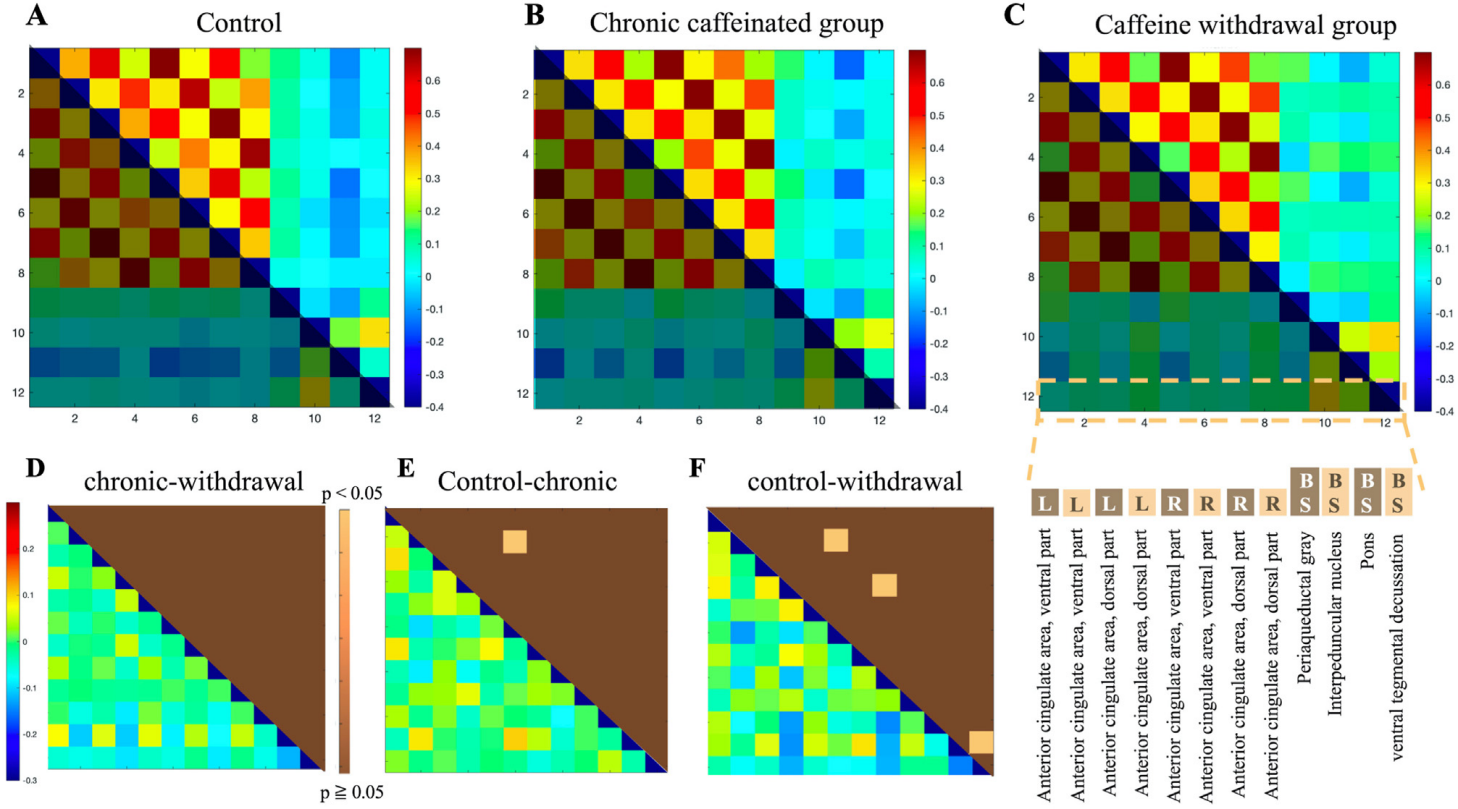
The matrix results of the functional connectivity analysis are shown (from left to right for the control (A), chronic caffeine (B), and caffeine withdrawal group (C)). The vertical and horizontal axes represent the hippocampus and DG extracted from 336 brain regions. The lower triangle of each data has different colors because the data are similar. In the lower part (From D to F), as in Figure 3, the correlation coefficient significance analysis (upper triangle) results and the subtraction of the absolute value of the correlation coefficient (lower triangle) are presented in the bottom row. The details of the extracted region names are shown in the lower right corner. The details of the area names are depicted in the lower right corner (from left to right, ACC of left brain, ACC of the right brain, and brain stem).

As shown in Figure 4, the ACC and SCC exhibited more regions of negative correlation in the control group. Blue areas with correlation coefficients of −0.3 to −0.2 in the control group turned green with correlation coefficients of 0.1–0.2 in the caffeine withdrawal group, suggesting that more areas exhibited more positive correlations. Similarly, the caffeine group exhibited more brain activity than the control group. In addition, we assessed the neural activity in the ACC and brainstem regions (GM around the pons, suture nucleus, and ventral tegmentum), with a total of 12 regions. As shown in Figure 5, the brain correlation coefficient between the ACC and brainstem showed that the caffeine withdrawal group had the most activity. However, based on the p-values between the chronic caffeinated and caffeine withdrawal groups, the control and chronic caffeinated groups, and the control and withdrawal caffeine group, only a small area had significant differences. Therefore, the effect of caffeine on brain activity was not as significant as that in the ACC and SCC.

### 3.4 Neural activity in the hippocampus and dentate gyrus in the matrix analysis

**Figure 6:**
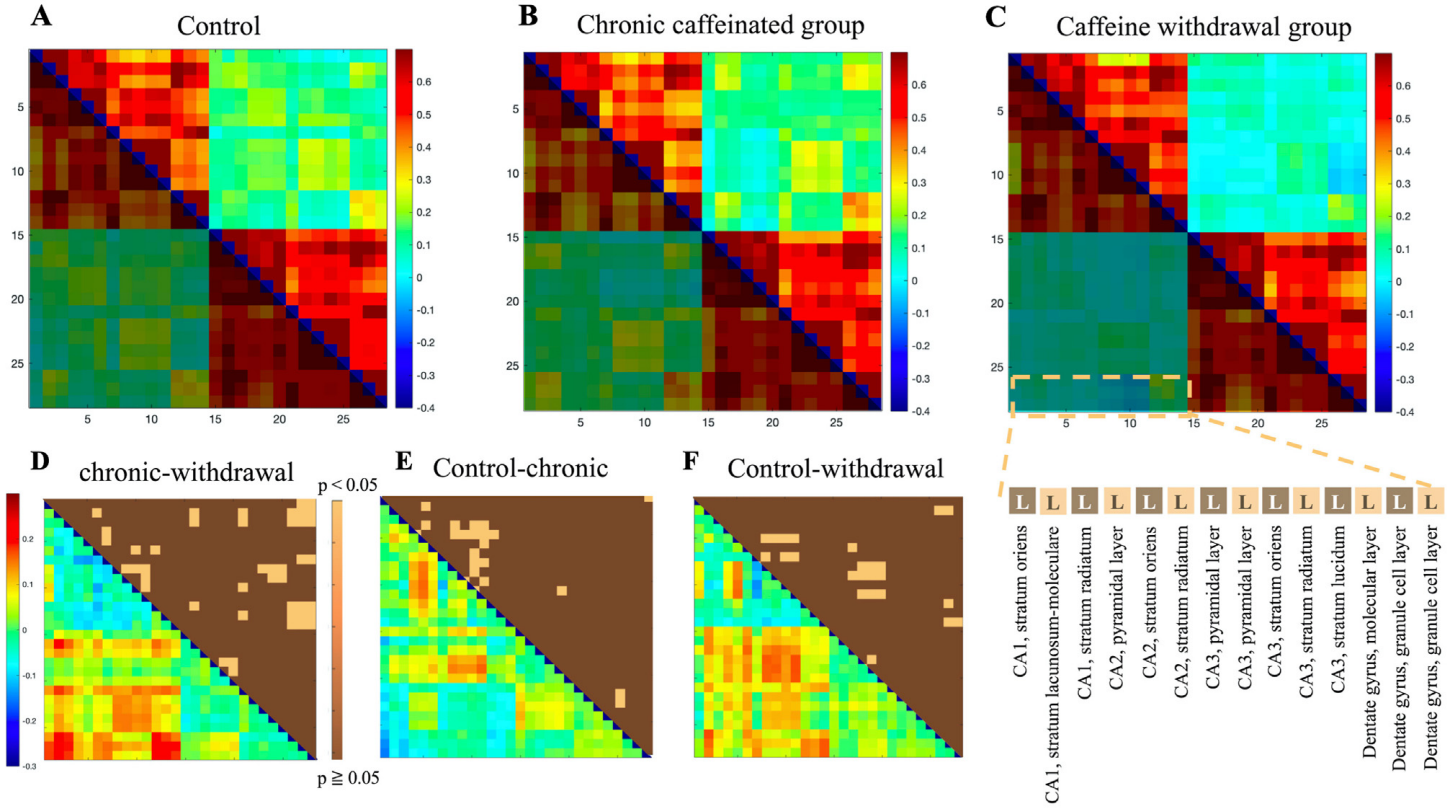
The matrix results of the functional connectivity analysis are shown in the upper row (from left to right for the control (A), chronic caffeine (B), and caffeine withdrawal groups (C)). The vertical and horizontal axes represent the hippocampus and DG extracted from 336 brain regions. The lower triangle of each data has different colors because they are identical. In the lower part (From D to F), as in Figure 3, the correlation coefficient significance analysis (upper triangle) results and the subtraction of the absolute value of the correlation coefficient (lower triangle) are presented in the bottom row. The details of the extracted region names are shown in the lower right corner. R is omitted because it has the same region name and the same order as L.

Next, we assessed the activity in the hippocampal region and dentate gyrus (DG), with a total of 14 regions. In the lower part of Figure 6, the left hippocampal C3 region of the brain showed significant changes in the p-values for comparing the control group and the chronic caffeine group. The neural activity in the left and right sides of the DG decreased in the caffeine withdrawal group.

The neural activity in the left and right sides of the DG decreased in the caffeine withdrawal group.

The negative correlation between the right and left hemispheres was significantly enhanced in the caffeine withdrawal group compared with the control and chronic caffeinated groups. The strength of the ipsilateral connection increased, and the contralateral connection further showed a negative correlation.

### 3.5 Neural activity in the ACC in the matrix analysis (brain network correlated with stress)

**Figure 7:**
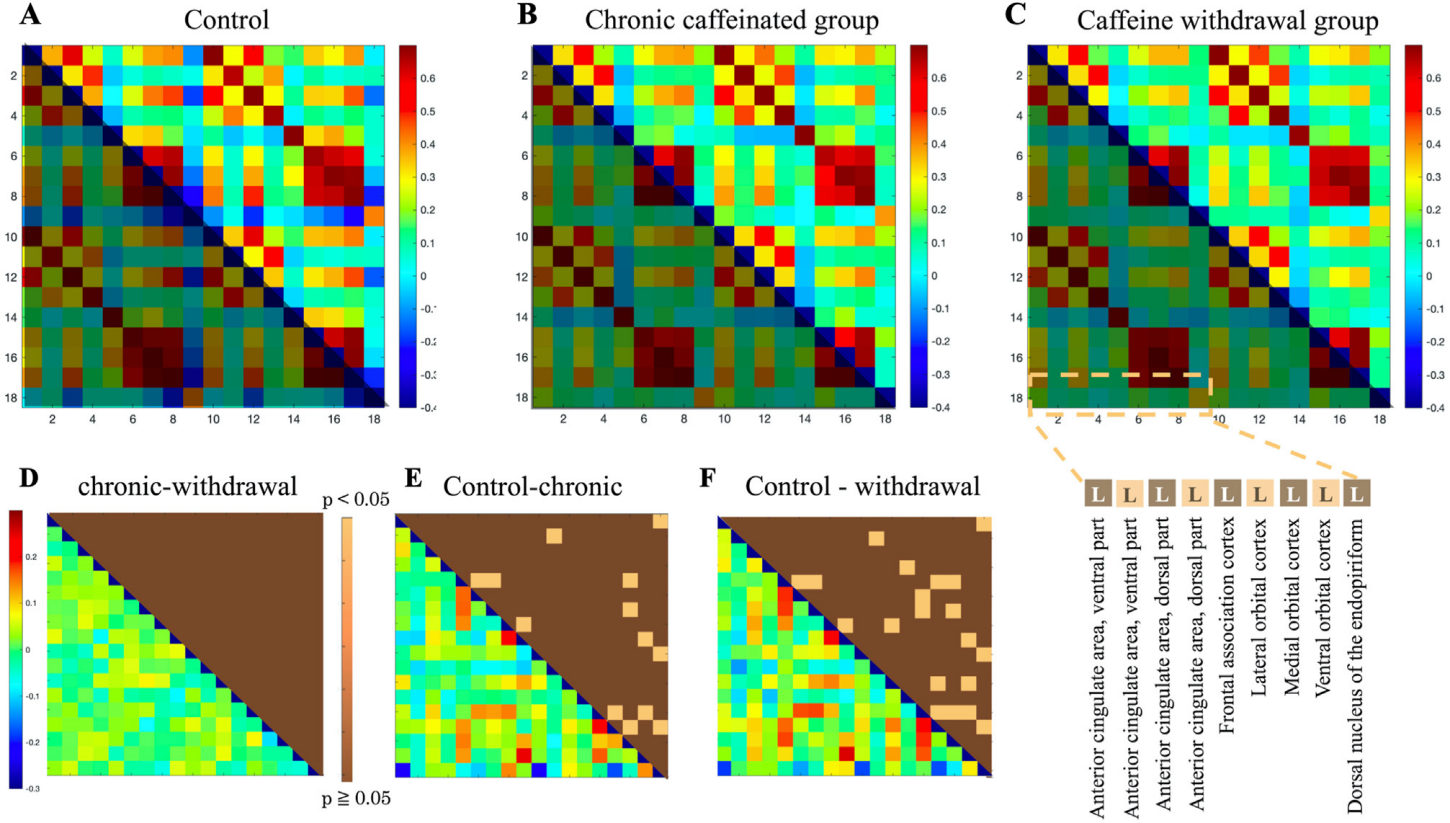
The matrix results of the functional connectivity analysis are shown in the upper row (from left to right for the control (A), chronic caffeine (B), and caffeine withdrawal groups (C)). The vertical and horizontal axes represent the ACC–PFC extracted from 336 brain regions. The lower triangle of each data has different colors because they are identical. In the lower part (From D to F), as in Figure 3, the correlation coefficient significance analysis (upper triangle) results and the subtraction of the absolute value of the correlation coefficient (lower triangle) are presented in the bottom row. The details of the extracted region names are shown in the lower right corner. R is omitted because it has the same region name and the same order as L.

Figure 7 shows the activity in the ACC, with a total of nine regions in the prefrontal cortex (PFC). With a focus on the frontal ACC and other prefrontal regions, the control group had a strong positive correlation. In contrast, there was no significant difference when comparing the chronic caffeinated and caffeine withdrawal groups. A negative correlation was observed in the dorsal nucleus of the endopiriform (EPN) in the control group. However, a positive correlation was noted in the chronic caffeine and caffeine withdrawal groups.

### 3.6 Neuronal activity of the olfactory bulb in the matrix analysis

**Figure 8:**
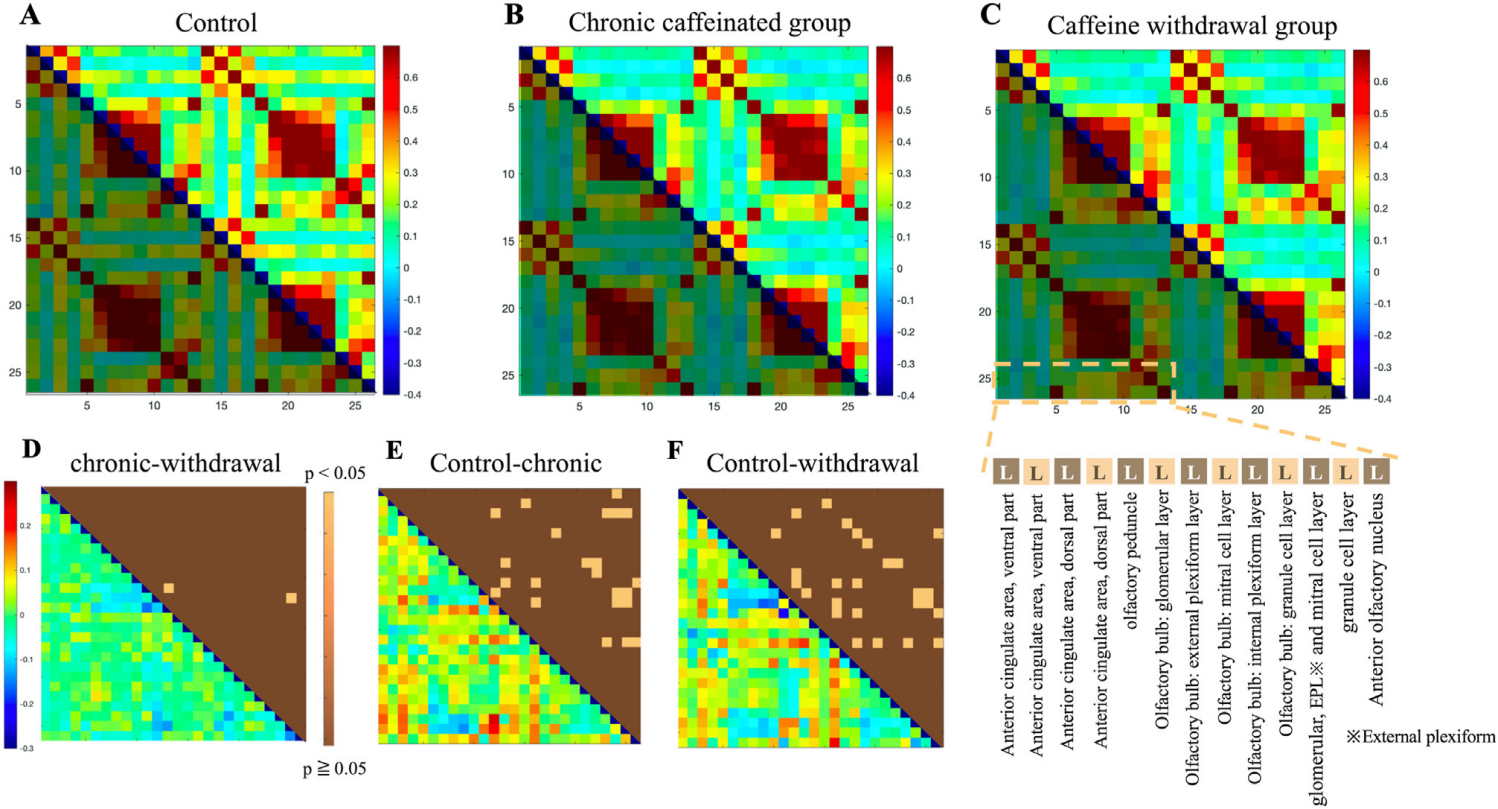
The matrix results of the functional connectivity analysis are shown in the upper row (from left to right for the control (A), chronic caffeine (B), and caffeine withdrawal groups (C)). The vertical and horizontal axes represent the OB–ACC extracted from 336 brain regions. The lower triangle of each data has different colors because they are identical. In the lower part (From D to F), as in Figure 3, the correlation coefficient significance analysis (upper triangle) results and the subtraction of the absolute value of the correlation coefficient (lower triangle) are presented in the bottom row. The details of the extracted region names are shown in the lower right corner. R is omitted because it has the same region name and the same order as L.

Figure 8 shows the neuronal activity in 13 regions of the olfactory bulb (OB) and ACC.

Compared with the control group, the chronic caffeine group and caffeine withdrawal group showed an overall decrease or shift to an uncorrelated state of OB and ACC brain neural activity.

The p-values also showed that there are many areas where significant differences when comparing the control group with the chronic caffeine and caffeine withdrawal groups.

## 4 Discussion

Caffeine is frequently used as a psychoactive substance that stimulates the central nervous system and provides various benefits, but specific cranial nerve network activation by caffeine administration remains unclear. In addition, caffeine withdrawal may cause headaches after approximately 24 h among chronic caffeine consumers. Therefore, this study assessed neural activity using a noninvasive technique, namely rs-fMRI, to specifically evaluate caffeine-induced changes in brain activity in mice. The neural activity of the control, chronic caffeinated group, and caffeine withdrawal groups were analyzed via ICA and functional matrix analysis.

### 4.1 Resting-state network analysis via ICA

Regarding the control and chronic caffeinated groups, the results revealed a network similar to the DMN observed in a previous study [34]. However, in the caffeine withdrawal group, a neural network was observed around the temporal lobe, but we did not observe a DMN-like network similar to previous studies. There were also differences on the left and right sides of the extracted networks. A previous study showed decreased functional connectivity in the DMN among patients with migraine [35]. Moreover, according to other research, a low frontoparietal network, which is one of the functional disconnections in migraine, has been observed [36]. In addition, asymmetric activation and impairment of neural networks in the right and left hemispheres have been observed in stress-related mental disorders [37].

In the current study, the caffeine withdrawal group presented with left and right asymmetry network, indicating that stress load and migraine-like neural activity were caused by caffeine withdrawal.

Regarding major networks other than the DMN, a network similar to the lateral cortical network (LCN) was observed in all groups. The LCN reportedly has a role similar to that of the human central executive network, which has been reliably identified in studies using seed-based correlations in rodents [34]. A network similar to the LCN network was observed in all groups in the present study. The ventral midbrain is a network that includes the amygdala, hypothalamus, and ventral tegmental area, and it was not identified in the caffeine withdrawal group. Because the causal relationship between caffeine withdrawal and reduced ventral midbrain network activity is unclear, further investigation is warranted.

### 4.2 Neural activity of the whole brain

Caffeine has a stimulating effect, and caffeine intake reduces reaction time for tasks correlated with attention and working memory and improves accuracy in numerical calculations [38].

There are more regions with significant differences in the control group/chronic caffeinated group in this study. Administration of caffeine increases the activation area of the brain over a wide range.

### 4.3 Neural activity in the ACC/SCC/brainstem

The ACC is a major region of the central pain processing network, with increased activity in the ACC region during migraine attacks. The SCC is the final cortical input to the pain system and is involved in the fine-grained processing of pain intensity and quality [39, 40]. The current study examined the activation of brain activity of the chronic caffeinated and caffeine withdrawal groups. In addition, brain regions with significant differences increased when comparing the control group to the caffeine withdrawal group. Hence, there was more neural activity correlated with pain processing in the caffeine withdrawal group than in the control group. In addition, previous studies have shown that the ACC and the cerebral cortex and brainstem are involved in pain processing [41].

Moreover, the caffeine withdrawal group had a higher neural activity in the pons, ventral tegmental area, and ACC than in the chronic caffeinated group. Caffeine withdrawal might have increased neural activity in the ACC–SCC and ACC–brainstem, thereby indicating that neural activity enhancement occurred in the brain similar to that in migraine.

There are also reports that caffeine pretreatment blocks adenosine, leading to prevention of vasodilation, which is considered to be one cause of migraine [42]. Adenosine A2A receptors have also been suggested as potential targets for migraine treatment [42]. In the present study, the chronic caffeine group exhibited no neural activity in the pain-related ACC–SCC and ACC–brainstem networks. We believe that the effects of caffeine administration on migraine in humans should be investigated using rs-fMRI network analysis.

### 4.4 Neural activity in the hippocampus and DG

There was a significant difference in neural activity in the left side of the hippocampus between the control and chronic caffeinated groups. This might be attributed to the left–right difference in the reception of hippocampal synapses. There is a difference in the size of synapses on the left and right sides of the hippocampus that receive signals from the ipsilateral and contralateral sides. Further, the neurotransmission is asymmetrical [43]. The effects of caffeine on the activity in the left and right hippocampus should be evaluated in detail from the perspective of neurotransmission.

Moreover, another study showed that 4-week caffeine treatment had protective effects against sleep disorder-induced early long-term potentiation damage in the DG region [44]. Calmodulin-dependent protein kinase II, which mediates signal transduction in memory and learning, was significantly increased by caffeine administration [44]. The caffeine concentration used in the previous study was similar to that in the current one (0.3 mg/mL), and the activation of an area in the DG region was also observed in the chronic caffeine group. In other previous studies, immunohistochemistry of brain sections from mice [45] and orthogonal omics analysis of molecular pathways involved in neural processing demonstrated that caffeine administration alters neural progenitor cell proliferation and neurotransmitter metabolism [6]. In the present study, we were unable to clarify the effects of caffeine administration on neuronal proliferation and neurotransmission because we focused on imaging analysis. It is a future goal to investigate the effects of caffeine on the brain in an integrated manner from the viewpoint of neural networks and neuronal cytology.

However, there are cases in which hippocampus-related memory performance is reportedly deficient in rodents that chronically consume caffeine. For example, a caffeine intake concentration of ≤1.0 mg/mL for 3 weeks affects the sleep cycle and slows hippocampal activity [46]. Neural enhancement in hippocampal regions involved in learning and memory may depend on the caffeine concentration. Furthermore, there was an enhanced positive correlation between bilateral neural activity and a negative correlation between the contralateral neural activity in the caffeine withdrawal group. More contrast was observed in the images of the caffeine withdrawal group compared with the chronic caffeinated group.

Previous studies have shown that pain vigilance is associated with enhanced hippocampal neural activity [47] and that people are more sensitive to this pain, similar to migraine. In addition, it is thought that when the brain is in an uncomfortable state, adenosine A2A receptors (A2ARs) become hyperactive [48, 49] and A2ARs are most abundant in hippocampal nerve endings [50]. Therefore, the enhanced pain-related neural activity that occurs during caffeine withdrawal may also affect brain function in the hippocampal region.

### 4.5 Neural activity in the ACC and PFC

The ACC is involved in emotional cognition, which controls emotional responses to stimuli and selects appropriate actions. Moreover, this region is sensitive to stress [37]. In addition, a number of stress-loading experiments reduced the volume of ACC and PFC, as well as the atrophy of the dendritic tips and the number of branches in pyramidal cells [37]. Similarly, some reports showed decreased brain function in the PFC of depressed mice [51]. In this study, the neural activity in the PFC decreased, and the there was a significant difference in several areas around the prefrontal cortex. Furthermore, there was no significant difference between the chronic caffeinated and the caffeine withdrawal groups. Therefore, caffeine intake might affect the sleep cycle and cause stress due to continued wakefulness. On the other hand, it has been reported that the average concentration of caffeine consumed by coffee drinkers restored the decrease in synaptic transmission by regulation adenosine's A1R-mediated inhibition of excitatory synaptic transmission [52]. It is also noted that the effects of caffeine on excitatory synaptic transmission in humans are not readily observable in rodents, although a link has been suggested, it is not possible to conclude that caffeine administration has reduced cranial nerve function in the prefrontal cortex in general.

In addition, the neural activity of the EPN was enhanced in the chronic caffeinated and caffeine withdrawal groups. EPN mediates the neural activity of the basolateral amygdala, which is involved in memory formation correlated with anxiety and stress [53]. It is regulated to integrate information associated with fear and stress caused by external stimuli and to channel memory information to the amygdala and hippocampus via the EPN [47]. The results of this study regarding brain neural activity showed that caffeine administration and withdrawal resulted in neural activity similar to that seen when mice were stressed. However, there is substantial ambiguity regarding caffeine administration and stress. The following section will introduce some of the previous studies that reported contradictory findings to those of this study.

### 4.6 Contradictory effects of caffeine administration on the brain

The current study suggested that caffeine administration enhances neural activity in stress-related brain regions and that caffeine intake induces activation of stress- and fear-related brain functions in mice. However, there is ambiguity regarding caffeine administration and stress. Reportedly, ingestion of caffeine, an adenosine receptor antagonist, might be a prophylactic anti-stress strategy to normalize synaptic function and stabilize mood-related behavioral changes via A2AR blockade [50].

The current study found that EPN neural activity was enhanced in the chronic caffeine intake and caffeine withdrawal groups. Conversely, adenosine receptors in the amygdala regulate the amygdala circuitry in fear memory, and it has been reported that caffeine, an antagonist of adenosine, blocks adenosine A2ARs, which lessens fear memory relative to that observed in controls [54].

However, there are reports that stress from disrupted sleep patterns caused by high doses of caffeine in mice is associated with suppression of neurogenesis [45] and that both acute caffeine consumption in humans and long-term caffeine consumption in rodents results in anxiety tendencies, leading to inconsistent effects of caffeine reported in the conclusions of some studies [11].

In addition, one previous study reported that in an fMRI study on humans, a group of habitual coffee drinkers showed reduced functional connectivity in networks involving subcortical and hindbrain regions associated with somatosensory, motor, and emotional processing [12]. This finding is similar to the results reported in the current study and supports the idea that chronic caffeine administration leads to decreased brain function connectivity around the prefrontal cortex.

It has also been reported that the reduction in brain neural networks correlates with the frequency of consumption of caffeinated products and that this change is reproduced even when a single cup of coffee is consumed by people who do not habitually drink caffeine, suggesting a possible causal relationship between coffee consumption and changes in network connectivity in the brain [12].

Thus, the effects of caffeine administration on the brain are complex, and they depend on the concentration and duration of administration. It would be interesting to examine in detail the mechanism by which the brain is affected by the duration, concentration, and timing of caffeine administration.

### 4.7 Neural activity in the OB and ACC

The OB is activated by the ciliary body and the olfactory entorhinal cortex, in which the activity is secondary to the amygdala and other brain regions such as the hippocampus, ACC, and the orbitofrontal cortex [55].

Olfactory neural networks affect the brain regions responsible for emotion. Previous studies have shown that disturbances in olfactory neural circuits can affect limbic system function and interfere with emotional processing in mice [55].

The current study showed that caffeine intake decreased the neural activity in the OB and ACC. A previous study using a mouse model of agitated depression induced by bilateral olfactory bulbectomy reported that caffeine administration prevented neurodegeneration of the striatum and pisiform cortex of the OB and averted hyperactivity and recognition memory deficits [56].

As noted in Section 4.6, some inconsistencies can be found regarding the effects of caffeine administration on the brain. The results of this study suggest that the OB experienced decreased neural activity and stress. The results also showed little change in the number and location of regions, with a significant difference in the caffeine withdrawal group compared with the chronic caffeine group. Therefore, the decrease in neural activity between the ACC and OB could be maintained after 24 h of caffeine withdrawal.

Thus far, we have discussed the deleterious effects caused by caffeine intake. However, there are also reports showing the benefits of caffeine intake. Parkinson’s disease is a movement disorder caused by a deficiency in dopamine in the basal ganglia. In the Parkinson’s disease model, a neurotoxin, referred to as methyl phenyl tetrahydropyridine (MPTP), was used, and it was found that it caused degeneration of dopaminergic neurons. The neural function of the OB, cerebellum, and cortical regions is preserved via administration of caffeine prior to MPTP [47, 57, 58].

Thus, caffeine affects brain activity from a neuroprotective perspective. This study assessed healthy mice. However, it is also interesting to examine the effects of caffeine in diseased mice, such as that in a Parkinson’s model. The withdrawal time changes after long- and short-term withdrawal should also be examined. Moreover, alterations in brain activity according to different concentrations of caffeine and the neurological activity of the brain if caffeine is administered acutely via the abdominal cavity must be examined.

The current study examined changes in neural activity caused by caffeine intake in specific regions. The results suggested that caffeine, a commonly consumed ingredient, can cause a variety of changes in brain activity, highlighting its potential for medical purposes.

## 5 Conclusion

Caffeine has various benefits. It can improve alertness, delay the onset of neurological diseases, such as Alzheimer’s disease, and slow the decline of cognitive abilities caused by aging. However, caffeine also causes withdrawal symptoms and addiction.

In the current study, the neural activity of specific brain regions changed after chronic caffeine administration and withdrawal. In particular, caffeine administration was associated with significant stress-related neural activity, suggesting that the stress response was enhanced by the arousing effects of caffeine. In addition, there was enhanced cranial nerve activity correlated with headache and changes in specific cranial nerve networks in the OB. These results were correlated with those of previous studies on brain changes from a histological perspective.

## Declarations

### Author contribution statement

Mitsuki Rikitake: Conceived and designed the experiments; Performed the experiments; Analyzed and interpreted the data; Wrote the paper.

Sachiko Notake; Karen Kurokawa: Conceived and designed the experiments; Performed the experiments.

Junichi Hata: Conceived and designed the experiments; Performed the experiments; Analyzed and interpreted the data; Contributed reagents, materials, analysis tools or data.

Fumiko Seki; Yuji Komaki; Naoki Kawaguchi; Yawara Haga: Analyzed and interpreted the data.

Hinako Oshiro: Performed the experiments.

Daisuke Yoshimaru; Ken Ito; Hirotaka James Okano: Contributed reagents, materials, analysis tools or data.

### Funding statement

Associate Professor Junichi Hata was supported by 10.13039/100019808MRI platform [JPMXS0450400021], 10.13039/501100001691Japan Society for the Promotion of Science KAKENHI [JP20H03630].

### Data availability statement

The data that has been used is confidential.

### Declaration of interest’s statement

The authors declare no conflict of interest.

### Additional information

No additional information is available for this paper.
